# Undergraduate nursing students’ post-coronavirus disease 2019 academic learning experiences at a public university in South Africa: A qualitative study

**DOI:** 10.4102/hsag.v30i0.3240

**Published:** 2025-11-21

**Authors:** Claudine H. Wittenschinskey, Wilma ten Ham-Baloyi, Mercia Kramer

**Affiliations:** 1 Department of Nursing Science, Faculty of Health Sciences, Nelson Mandela University, Gqeberha, South Africa

**Keywords:** nursing education, COVID-19 pandemic, undergraduate students, university, academic learning experiences, South Africa

## Abstract

**Background:**

The coronavirus disease 2019 (COVID-19) pandemic disrupted nursing education globally, affecting theoretical instruction, clinical training and students’ well-being.

**Aim:**

To explore and describe undergraduate nursing students’ academic learning experiences in the post-COVID-19 period at a South African public university.

**Setting:**

A public university in South Africa.

**Methods:**

A qualitative, exploratory-descriptive, and contextual design underpinned by Bronfenbrenner’s ecological theory was applied. Twenty-nine purposively selected undergraduate nursing students participated in four face-to-face semi-structured focus group discussions facilitated by an independent fieldworker. Data were thematically analysed by an independent coder.

**Results:**

Three themes emerged. Firstly, students reported persistent challenges following the return to in-person learning, including misalignment between teaching and assessment methods, gaps in clinical exposure, and heightened mental health strain. Secondly, enablers of academic success were identified, such as self-directed learning, peer support, and personal and professional development. Thirdly, participants suggested improvements to post-pandemic learning, including curriculum reform, more flexible timetabling, and safer, better-resourced clinical placements.

**Conclusion:**

The post-pandemic academic environment continues to shape nursing students’ learning and well-being. While students demonstrated resilience through self-regulation and peer collaboration, institutional reforms remain essential to foster academic success and professional readiness.

**Contribution:**

The study provides context-specific insights into post-pandemic nursing education in South Africa and highlights the importance of responsive, student-centred strategies.

## Introduction

The coronavirus disease 2019 (COVID-19) pandemic, declared a global health emergency by the World Health Organization (2020), disrupted educational systems worldwide, including nursing education at universities. Caused by the Severe Acute Respiratory Syndrome Coronavirus 2 (SARS-CoV-2 virus), this unprecedented crisis forced universities globally to rapidly adapt teaching and learning modalities under extraordinary circumstances, as noted by Mishra et al. (2020). As of 2024, the world has largely transitioned into the post-pandemic phase characterised by reduced infection rates and eased restrictions; yet, the consequences of the educational disruptions continue to unfold (Martin et al. 2023).

During the pandemic, universities shifted from traditional face-to-face classroom teaching to online and blended learning approaches, redefining the ‘new normal’ in nursing education (Kim & Kim 2023). This transition, while necessary, introduced significant challenges such as inadequate access to technological resources and disruptions in clinical placements, especially for students from disadvantaged backgrounds, according to Mashayisa and Ivala (2022). Conversely, the move to online platforms enhanced self-directed learning (SDL), student confidence and access to educational materials, creating opportunities to improve future learning outcomes, as observed by Molato and Sehularo (2022).

Globally, nursing students experienced varied difficulties adjusting to virtual learning environments, including poor internet connectivity, insufficient orientation to new learning tools, and increased academic anxiety (Ironsi 2022). These challenges were compounded by mental health concerns such as isolation and stress, highlighting the urgent need for psychological support services within universities (O’Dea & Stern 2022). In South Africa, infrastructural barriers like power outages and limited Information and Communication Technology (ICT) access further hindered the effective transition to online education, particularly in rural areas, as highlighted by Mtshali and Zwane (2019). The institution where this study was conducted faced similar constraints, with intermittent connectivity and varying levels of student digital literacy, all of which affected the consistency and quality of online learning and assessment during and after the pandemic.

The pandemic also exposed critical limitations in delivering clinical education, a cornerstone of nursing training. Many students reported reduced hands-on practice and supervision during clinical placements, negatively affecting competency development and professional confidence (Michel, Ryan & Mattheus 2021). In South Africa, these challenges were compounded by restricted access to clinical facilities, staffing shortages and safety concerns during community placements (Baloyi, Jarvis & Mtshali 2022; Fadana & Fember 2021). This loss of experiential learning poses risks to the readiness of new graduates entering healthcare systems (Maboe & Bezuidenhout 2022). At the study institution, disruptions in placement schedules and limited opportunities for direct patient care further constrained experiential learning, raising concerns about the clinical readiness of new graduates entering the workforce.

Despite these challenges, the post-pandemic era offers an opportunity to integrate the strengths of both traditional and digital teaching methods to enhance nursing education. Understanding undergraduate nursing students’ academic learning experiences in this context is essential to inform student support services, curriculum development and future contingency planning (Dean & Campbell 2020). While international research has highlighted these issues, there remains a notable gap in knowledge regarding South African university nursing students’ post-COVID-19 academic experiences.

This study aimed to explore and describe undergraduate nursing students’ academic learning experiences in the post-COVID-19 period at a South African public university.

## Research methods and design

### Research design

This study employed a qualitative, exploratory-descriptive, and contextual research design. The approach was selected to gain an in-depth understanding of nursing students’ experiences in the post-COVID-19 era. Bronfenbrenner’s ecological systems theory (Bronfenbrenner & Morris 2006) underpinned the research framework, providing a structured lens through which to analyse the impact of various environmental factors on students’ learning.

### Setting

The study was conducted at a public university in the Eastern Cape, South Africa. This university offers the Bachelor of Nursing and Midwifery programmes. The institution has infrastructure conducive to hybrid learning, including free Wi-Fi, electronic devices (e.g. laptops via bursaries) and monthly data allocations for students.

### Participants and sampling

The target population comprised undergraduate nursing students enrolled in Bachelor of Nursing programmes, including the R174 Bachelor of Nursing and Midwifery and the R425 Nurse (General, Psychiatric, Community) and Midwife programmes. Students were drawn from two campuses: 1st- and 2nd-year students attended one campus, while 3rd- and 4th-year students attended the other. A purposive sampling strategy was employed to recruit participants with relevant knowledge and experiences of post-COVID-19 academic learning. Inclusion criteria comprised registered students with a valid registration number enrolled in either programme across all year levels. Data collection took place in October 2023, by which time most countries had lifted major COVID-19 restrictions, with widespread vaccination and reduced hospitalisations, marking a post-pandemic period. In education, this ‘post-COVID-19’ phase reflects the resumption of in-person teaching and learning (Jacob & Stanojevich 2024), ensuring that all students have post-COVID-19 academic experiences.

Sampling continued until data saturation was reached. To achieve this, between two and five focus group discussions (FGDs) were planned, each comprising 8–10 participants (Hennink, Kaiser & Weber 2019), yielding a potential total of 16–50 participants. Data saturation was achieved after four FGDs involving 29 participants, when no new themes or insights emerged, indicating that the data sufficiently captured the experiences and perspectives of the target population.

### Recruitment

After obtaining ethical clearance and institutional permission, year-level coordinators – academic staff responsible for overseeing and supporting students within a specific year of the programme – facilitated recruitment by organising information sessions with students. As the researcher (first author) was a year-level coordinator and a trained fieldworker – independent of both the students and the institution – they conducted the face-to-face recruitment sessions. During the 15 min – 30 min recruitment sessions held in a lecture venue, the independent fieldworker provided both verbal and written information about the study’s topic, aims and purpose. Details regarding the researchers’ and supervisors’ contact information, the dates, times, and venue of the FGDs, participants’ rights, and the availability of psychological support following data collection were also shared. Emphasis was placed on the participants’ right to withdraw at any time, consistent with the ethical principle of respect for persons. Students who expressed interest were scheduled into FGDs based on their availability.

### Data collection

Data were collected through semi-structured FGDs conducted in English between August and October 2023. The FGDs were held in private classrooms on campus, with only participants, the fieldworker (second author) and the researcher (first author) present. The fieldworker was a trained qualitative researcher with prior experience in facilitating FGDs and held a postgraduate degree in health sciences. The researcher, who observed the sessions and managed logistical support, is a registered nurse and an academic staff member but had no teaching responsibilities for the participating students.

An interview guide was developed based on literature and Bronfenbrenner’s ecological systems theory (Bronfenbrenner & Morris 2006), covering themes such as the transition to online learning, emotional and academic impacts, and support needs. The interview guide was refined after the first FGD with 1st-year students, which served as the pilot interview. One minor editorial revision, such as the removal of a duplicate question, was made to the interview guide after the pilot interview. Pilot data were incorporated into the main analysis, as no major amendments to the interview guide were required, and the data provided valuable insights relevant to the study’s aim (Malmqvist 2019). Each session was audio-recorded using two devices to ensure backup. Non-verbal cues and group dynamics were captured in detailed field notes taken by the observer.

Focus group discussions lasted 45 min – 60 min and were conducted in a respectful, participant-centred manner. Written informed consent was obtained for participation and audio recording. Pseudonyms such as ‘participant 1’ were used during the interviews to ensure participant confidentiality. The fieldworker used open-ended questions from the interview guide – such as *‘Could you describe any challenges you have encountered that have affected your academic learning within the nursing programme following the COVID-19 pandemic?’, ‘What facilitates your academic learning experiences within the nursing programme post-COVID-19?’*, and *‘What recommendations could you offer to enhance your academic learning experiences within the nursing programme post-COVID-19?’* – and employed prompts to elicit detailed responses, ensuring that all participants could contribute fully. Data collection was conducted with attention to interviewer neutrality, participant comfort and transparency in the setting. No follow-up interviews needed to be conducted. Audio recordings were securely stored and accessible only to the research team.

### Data analysis

Audio recordings were transcribed verbatim using Otter.ai, a software for transcribing raw data, and the researchers verified the transcripts for accuracy prior to analysis. Thematic analysis was conducted by the first author manually following Braun and Clarke’s (2006) six-step framework: familiarisation, coding, theme development, review, definition and reporting. Transcripts were independently reviewed and analysed by the researcher and an independent coder to enhance credibility. The researcher and the independent coder then discussed and compared their interpretations to reach consensus on the identified themes. Field notes supplemented the transcripts to provide context and capture non-verbal cues.

### Trustworthiness

Trustworthiness was established using Lincoln and Guba’s criteria. Credibility was ensured through independent coding, member checking, and supervisory review. Dependability was supported by consistent procedures, an audit trail, and the use of the same guide and venue. Confirmability was enhanced through detailed documentation and peer review of decisions. Transferability was achieved by providing rich contextual descriptions. Authenticity was addressed by presenting diverse participant voices and experiences.

### Ethical considerations

Ethical approval was obtained at Nelson Mandela University from the University’s Research Ethics Committee (REC-H, Ref. H24-HEA-NUR-017). The study adhered to the Belmont Report (National Commission for the Protection of Human Subjects of Biomedical and Behavioral Research 1979)’s principles of respect, beneficence and justice. Participation was voluntary, and written informed consent was obtained from all participants after full disclosure of the study’s purpose, procedures, risks and their right to withdraw. Confidentiality was ensured through pseudonyms, secure data storage and private data collection settings. Owing to the group setting, full confidentiality could not be guaranteed, and participants signed a confidentiality agreement. Participant well-being was prioritised by minimising risks and offering free psychological support post-discussion. During interviews, refreshments were provided to ensure participant comfort, and no other incentives were offered. Recruitment was equitable, with all eligible students given an equal opportunity to participate.

## Results

### Participant profile

A total of 29 nursing students participated in the study, representing a diverse group in terms of demographics, with most coming from provinces outside the Eastern Cape, South Africa. All participants were fluent in English, between 19 and 27 years of age and included a mix of female and male students across the FGDs. The group spanned all year levels: seven first-year students took part in the pilot study, five third-year students participated in FGD 1, six fourth-year students in FGD 2, and eleven second-year students in FGD 3.

Analysis of the focus group data revealed three overarching themes that encapsulate nursing students’ experiences with academic learning in the post-COVID-19 era. These include:

- academic learning challenges;
- factors facilitating academic learning;
- recommendations for improved post-pandemic academic learning.

Each theme reflects the nuanced realities of nursing students adapting to evolving learning environments after the COVID-19 pandemic.

### Theme 1: Academic learning challenges

Participants reported significant academic challenges during and after the pandemic, with issues ranging from technological barriers and mental health struggles to academic burnout and misalignment between teaching and learning approaches.

Students described the online learning environment during the pandemic as both isolating and stressful, particularly for those in rural areas with poor connectivity:

‘It was difficult for me. I’m from the rural areas … we had no connectivity. So, we were struggling with Outlook and Microsoft Teams.’ (Participant 6, FGD1)

The return to in-person learning post-pandemic presented a different kind of difficulty, as many students struggled to keep up with increased academic workload and heightened expectations:

‘It was too hard to adjust.’ (Participant 7, FGD1)

Several participants highlighted a disconnect between theoretical instruction and clinical expectations, leading to feelings of unpreparedness:

‘We don’t get the opportunity to do certain things [*in clinical settings*] … and you just do not get to learn it.’ (Participant 26, FGD3)

In addition to academic issues, students reported emotional exhaustion and a lack of psychological support:

‘We are burning out. And no one wants that.’ (Participant 15, FGD2)

‘You just cry behind the screen, and you go through everything alone.’ (Participant 10, FGD1)

Participants experienced academic and emotional challenges during and after the pandemic, including technological barriers, burnout, and gaps between theory and clinical practice. Both online and in-person learning posed difficulties, highlighting the need for targeted academic and psychological support.

### Theme 2: Factors facilitating academic learning

Despite the challenges, participants also identified positive factors that supported their learning during and after the pandemic. These included SDL strategies, peer motivation, and stronger personal and familial relationships.

The shift in learning styles led many students to become more independent and reflective. Self-awareness and the ability to identify their own learning needs emerged as key:

‘I had to make it work. I got a chance to rediscover what I want to do.’ (Participant 17, FGD2)

Interaction with peers during face-to-face classes was viewed as beneficial for engagement and comprehension:

‘We get to interact in class with other people … get to ask questions.’ (Participant 8, FGD1)

Participants also spoke about gaining clinical insight through preparation and self-study:

‘I know what someone with diabetes would be like with an HGT of one … I know it’s a duty. It is an emergency.’ (Participant 11, FGD1)

Furthermore, students appreciated the unexpected benefit of improved family bonds during lockdown, which provided emotional stability and support:

‘It was good on the family side … learning how to bake together, cooking together … it was nice.’ (Participant 26, FGD3)

Despite the challenges, participants identified factors that supported their learning during and after the pandemic, including SDL, peer interaction, and strengthened personal and familial relationships. The shift in learning styles fostered independence, reflection, and clinical insight, while family support during lockdown contributed to emotional stability, highlighting the importance of both academic and psychosocial resources in student learning.

### Theme 3: Recommendations for improved academic learning

Participants offered constructive suggestions to address the learning challenges they experienced. Recommendations were made across curriculum delivery, support structures, transport, safety, and timetabling.

Many participants called for curriculum realignment, advocating for nursing-specific content delivery and continued access to online learning options:

‘Let’s keep an open mind … we can always have an alternative [*online classes*].’ (Participant 10, FGD1)

Transport to clinical sites emerged as a significant concern with students proposing improved coverage or reimbursement:

‘Getting to the clinics is spending nearly 100 rand … that’s too much.’ (Participant 26, FGD3)

Students also urged for better-managed academic schedules, including designated days for rest:

‘One day off–no class, no clinic–just one day for yourself. That would be nice.’ (Participant 3, Pilot FGD)

Extended library hours and quiet, conducive study environments were proposed to enhance academic productivity:

‘I study better at night … the library can be more conducive [*by being open at night*].’ (Participant 3, Pilot FGD)

Safety in clinical placements was strongly emphasised:

‘Care for us and our safety … we don’t want to be seeing hashtag justice for ….’ (Participant 26, FGD3)

Participants provided constructive recommendations to address learning challenges, including curriculum realignment, flexible access to online learning, improved transport to clinical sites, better-managed timetables, extended library hours, and enhanced safety during clinical placements. These suggestions underscore the need for practical, student-centred interventions to support academic success and well-being.

## Discussion

This study explored the academic learning experiences of undergraduate nursing students during and after the COVID-19 pandemic. This study offers valuable insights into nursing students’ academic learning experiences in the wake of the COVID-19 pandemic. The findings illuminate ongoing challenges in transitioning to post-pandemic education, highlight student-driven mechanisms for academic resilience, and point towards strategic improvements in curriculum and institutional support. These are discussed below according to the three central themes.

### Academic learning challenges

The transition back to face-to-face and blended learning environments revealed significant academic and wellness-related challenges for students. Many struggled with adapting to traditional instructional and assessment methods after prolonged remote learning, revealing a disconnect between pedagogy and learning preferences. This misalignment, particularly in clinical assessments, has been linked in the literature to increased academic anxiety and reduced student confidence (Carless-Kane & Nowell 2023). The diminished clinical exposure during lockdown compounded this problem, weakening students’ preparedness for hands-on care – an issue also reported by O’Dea and Stern (2022) and consistent with the South African Nursing Council’s emphasis on practical competency (Matoso 2018).

The demanding schedules and inflexible timetables described by participants contributed to high levels of stress and exhaustion. This aligns with findings by Ironsi (2022), who asserts that student fatigue is often the result of institutional rigidity rather than academic incapacity. Similarly, Urtug and Faydali (2018) highlight that poorly balanced academic demands can hinder student engagement and retention. These structural issues were further aggravated by logistical challenges, such as commuting between clinical sites and campuses – an obstacle particularly relevant in the South African context (Du Preez, Scrooby & Jacobs 2019).

Mental health and wellness also emerged as critical concerns. Students described emotional strain, social isolation and a lack of access to university support services. This echoes research by Fernández–Gutiérrez and Mosteiro–Díaz (2021), which associates student distress with insufficient institutional care. Ongoing disruptions in learning environments may cause prolonged emotional instability among nursing students if support structures are inadequate.

### Factors facilitating academic learning

Despite the difficulties, several factors emerged as enablers of academic success. The development of SDL capabilities was prominent among participants who adapted by taking ownership of their academic responsibilities. This is in line with Prifti (2022), who argues that SDL fosters essential skills such as initiative, goal-setting, and self-evaluation – attributes especially valuable in professional healthcare contexts. Cazan and Schiopca (2014) similarly regard SDL as foundational for sustaining motivation in nursing education.

Peer learning and academic collaboration also played a critical role in supporting student progress. The restoration of face-to-face peer interactions enabled informal knowledge sharing and academic motivation, which has been shown to improve comprehension and retention in other studies (Sharma et al. 2023). Gehreke, Schilling and Kauffeld (2024) similarly underscore that peer support acts as both an academic and emotional resource, bolstering student persistence.

Furthermore, students’ ability to link theory with clinical observations, particularly during post-pandemic placements, reflects growing professional awareness. This is consistent with Aryuwat et al. (2024), who argue that experiential integration of knowledge is essential for effective clinical reasoning. For some students, the pandemic period also facilitated personal growth and reflection, leading to improved goal clarity and professional commitment.

The value of familial emotional support was another notable finding. Literature confirms that strong social support networks, particularly during crisis recovery periods, can promote psychological resilience and improve academic performance (Cooper & Bronwell 2020).

### Recommendations for improved academic learning

Students offered suggestions to enhance their academic experiences, many of which resonate with existing best practices in nursing education. A key recommendation was curriculum reform that promotes flexibility, including the continued integration of online or hybrid learning. Lockee (2021) notes that blended learning not only increases accessibility but also accommodates diverse learning styles, making it a sustainable model for nursing education post pandemic.

Students also highlighted the need to address structural and logistical barriers, such as transportation costs and safety concerns related to clinical placements. These findings reaffirm the need for institutional investment in transport subsidies and secure placement planning, as recommended by Du Preez et al. (2019) and Doyle et al. (2017).

A recurrent suggestion was for more thoughtful academic scheduling. This includes incorporating recovery time between intensive clinical duties and lectures, which supports both cognitive performance and emotional well-being (Ertuğ & Faydali 2018). Students’ advocacy for longer library hours and better access to study environments also echoes findings by Kariippanon et al. (2019), who suggest that flexible academic infrastructure is integral to learning effectiveness.

Finally, participants’ concern about safety in clinical placements underscores the need for institutions to take a proactive role in protecting students. Ensuring physical safety during community placements and offering psychological debriefings post placement are consistent with global standards in nursing education risk management (Doyle et al. 2017).

The study findings highlight that nursing students faced considerable academic and emotional challenges during the transition back to face-to-face and blended learning post COVID-19. Despite these difficulties, factors such as SDL, peer collaboration, clinical integration, and family support facilitated their academic and professional development. Students’ recommendations – including flexible curricula, improved scheduling, transport and safety measures, and enhanced study resources – underscore the need for holistic, student-centred strategies to optimise learning and well-being in the post-pandemic educational context.

### Strengths and limitations

A key strength of this study is that the use of FGDs enabled deep insights into collective and individual perspectives, enhancing the authenticity and relevance of the findings. The inclusion of participants from multiple levels of study and different campuses adds to the diversity and transferability of the results.

However, limitations exist. The study was conducted within a single university context in South Africa, which may limit generalisability to other institutions or regions. As is common with a generic qualitative design, participants were self-selected, which may have introduced selection bias. Students with stronger opinions or more pronounced experiences could have been more likely to participate, potentially overrepresenting certain perspectives. First-year students mainly provided insights on post-COVID-19 experiences, while higher-year students were able to compare their experiences during and after the pandemic. Additionally, the dynamic nature of the pandemic and post-pandemic period means that findings represent a snapshot in time, and ongoing research is needed to track long-term educational outcomes.

### Implications and recommendations

The findings of this study suggest several actionable implications for universities, particularly nursing programmes. Universities should undertake curriculum reform by aligning courses with students’ learning preferences, incorporating flexible delivery methods such as hybrid and asynchronous formats, and diversifying assessment approaches. Strengthening well-being and support services is crucial, particularly for off-campus or clinical students, with peer mentorship programmes providing additional social and emotional support. Academic timetables should prioritise student well-being by including buffer days for recovery and staggering clinical and theoretical sessions to enhance engagement. Investments in infrastructure are needed to expand digital resources, improve virtual library access, and ensure physical safety and transportation during clinical placements. Lastly, actively incorporating student feedback into decision-making processes will help ensure academic policies are responsive and contextually relevant.

## Conclusion

This study highlights the complex interplay of challenges and adaptations that defined nursing students’ academic experiences during and after the COVID-19 pandemic. While students faced significant barriers – including limited clinical exposure, academic misalignment, burnout, and wellness concerns – they also demonstrated resilience through SDL, peer collaboration and personal growth.

These findings underscore the necessity for nursing education to evolve towards more flexible, student-centred models that prioritise holistic support, equitable access and professional readiness. As the higher education sector transitions into a post-pandemic era, it is imperative that institutions not only address the gaps exposed by the crisis but also harness the lessons learned to build more resilient, inclusive and future-oriented learning environments.
